# Alpha-defensin-dependent enhancement of enteric viral infection

**DOI:** 10.1371/journal.ppat.1006446

**Published:** 2017-06-16

**Authors:** Sarah S. Wilson, Beth A. Bromme, Mayumi K. Holly, Mayim E. Wiens, Anshu P. Gounder, Youngmee Sul, Jason G. Smith

**Affiliations:** Department of Microbiology, University of Washington, Seattle, WA, United States of America; Stony Brook University, UNITED STATES

## Abstract

The small intestinal epithelium produces numerous antimicrobial peptides and proteins, including abundant enteric α-defensins. Although they most commonly function as potent antivirals in cell culture, enteric α-defensins have also been shown to enhance some viral infections *in vitro*. Efforts to determine the physiologic relevance of enhanced infection have been limited by the absence of a suitable cell culture system. To address this issue, here we use primary stem cell-derived small intestinal enteroids to examine the impact of naturally secreted α-defensins on infection by the enteric mouse pathogen, mouse adenovirus 2 (MAdV-2). MAdV-2 infection was increased when enteroids were inoculated across an α-defensin gradient in a manner that mimics oral infection but not when α-defensin levels were absent or bypassed through other routes of inoculation. This increased infection was a result of receptor-independent binding of virus to the cell surface. The enteroid experiments accurately predicted increased MAdV-2 shedding in the feces of wild type mice compared to mice lacking functional α-defensins. Thus, our studies have shown that viral infection enhanced by enteric α-defensins may reflect the evolution of some viruses to utilize these host proteins to promote their own infection.

## Author summary

Enteric α-defensins are an ancient form of host defense against pathogens, but until recently there was no robust *in vitro* system available to study their functions upon secretion from the cells that produce them naturally *in vivo*. Here, using small intestinal enteroids as a source of naturally secreted α-defensins, we show that enteric viral infection is increased in the presence of these peptides. We then show that α-defensins also enhance infection *in vivo*, as predicted by the results of the enteroid model. This study points to a new role for enteric α-defensins in promoting viral infection and has implications for the ability of these peptides to dictate viral tropism in the gastrointestinal tract.

## Introduction

Enteric α-defensins are effector peptides of the innate immune system that are abundantly expressed in both the human and mouse small intestine. Produced exclusively by specialized epithelial cells called Paneth cells, enteric α-defensins are broadly antimicrobial against bacterial and viral infections [1–3]. In humans, the enteric α-defensin repertoire consists of human defensin 5 (HD5) and HD6, while mice have undergone an expansion of their enteric α-defensin locus and encode upwards of 20 enteric α-defensins, termed cryptdins.

We have observed viral species-specific effects of purified α-defensins on human adenovirus (HAdV) infection in cell culture. For most species of HAdV, infection is potently neutralized, while infection by serotypes of HAdV-D and F is either resistant to enteric α-defensins or moderately enhanced [4]. Which of these phenotypes occurs in the presence of naturally secreted α-defensins has not been studied. To address this issue, we took advantage of our previously described small intestinal enteroid infection model [5].

Enteroids are an adult intestinal stem cell-derived primary culture system that models the intestinal epithelium by forming self-organizing “mini-guts” containing all of the major epithelial cell types of the small bowel including enterocytes, goblet cells, Paneth cells, and enteroendocrine cells [6]. We and others have shown that Paneth cell contents, including α-defensins and lysozyme, are constantly released and accumulate in the lumen of small intestinal enteroids to bactericidal levels [5, 7]. An important control is provided by enteroids from *Mmp7*^***-/-***^ mice, which lack functional α-defensins due to the absence of pro-defensin processing by matrix metalloproteinase 7 (MMP7) [5, 8]. Thus, comparison of viral infection in wild type and *Mmp7*^***-/-***^ enteroids affords an opportunity to examine the specific effects of naturally secreted α-defensins.

Since HAdVs do not replicate in mouse cells, we examined infection by two natural pathogens of mice, mouse adenovirus 1 (MAdV-1) and MAdV-2, in mouse enteroids. MAdV-1 infects macrophages and endothelial cells *in vivo* and causes acute encephalitis in infected mice, while MAdV-2 is naturally tropic for the small intestine and causes no overt disease [9]. Upon infection of mouse small intestinal enteroids, MAdV-2 but not MAdV-1 was able to replicate, consistent with their *in vivo* tropisms. Strikingly, more cells were infected and more MAdV-2 was produced from wild type enteroids compared to enteroids from *Mmp7*^***-/-***^ mice, demonstrating that naturally secreted α-defensins can enhance viral infection. Furthermore, increased fecal shedding was observed from wild type mice compared to *Mmp7*^***-/-***^ mice after oral infection with MAdV-2. Thus, the results from enteroid culture experiments predicted the phenotype of the infected mice. Finally, we show that purified mouse enteric α-defensins enhanced entry and infection by MAdV-2 in a receptor-independent manner. Therefore, our studies demonstrate that enhanced infection due to α-defensins, which was previously only observed in traditional cell culture, also occurs under physiologic conditions, leads to increased infection and viral shedding *in vivo*, and establishes α-defensins as a previously unknown host factor that contributes to viral tropism and promotes enteric viral infection.

## Results

### MAdV-2 but not MAdV-1 replicates in mouse small intestinal enteroids

**Fig 1:**
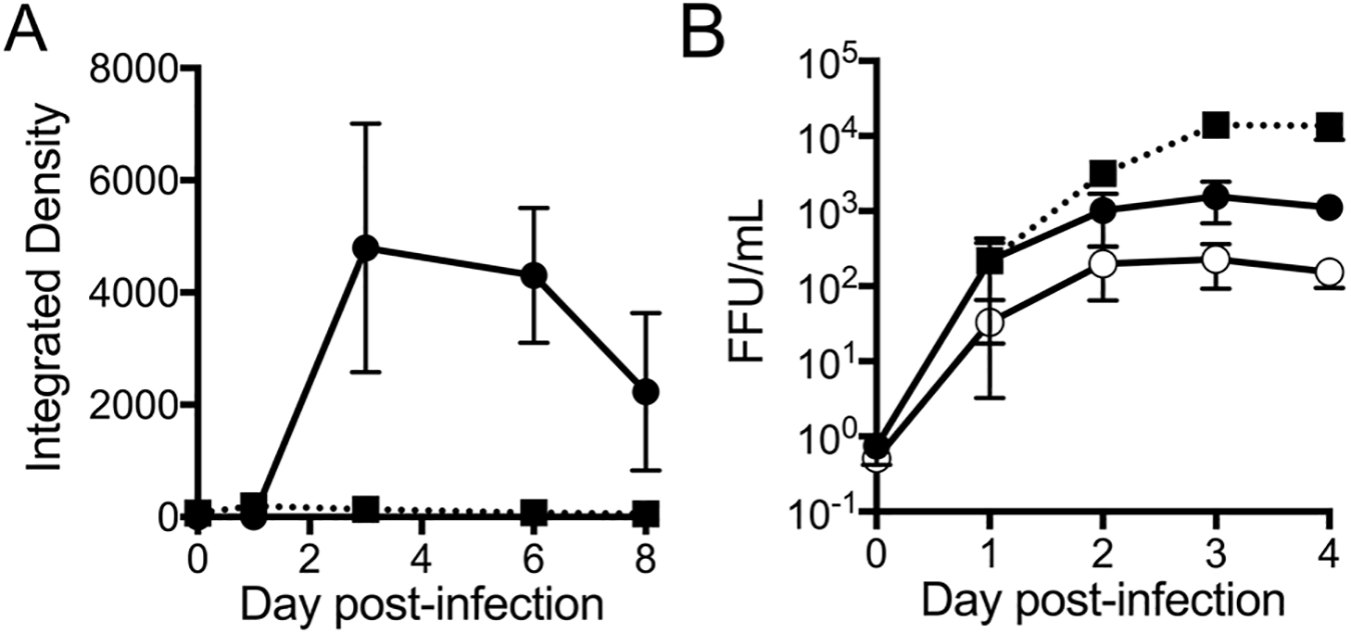
MAdV-2 but not MAdV-1 productively infects mouse small intestinal enteroids. Parallel cultures of (A) enteroids from C57BL/6 mice or (B) CMT-93 cells were infected with wild type MAdV-1 (closed squares, dotted line), MAdV-2 (closed circles, solid line), or MAdV-2.IXeGFP (open circle, solid line, CMT-93 cells only). Titers of progeny virus from samples harvested on the indicated days were determined on CMT-93 cells. For A, a single well was infected at each time point, and the integrated density of the immunofluorescence signal of the well is depicted. For B, the concentration of virus was calculated in fluorescence forming units (FFU) per mL from the TCID_50_ for each sample. Data are the average of 2 (A) or 3 (B) independent experiments ±SD.

To use enteroids to study the effect of α-defensins on adenoviral infection, we first determined whether or not MAdV-1 and -2 are able to replicate in these cultures. Enteroids from C57BL/6 mice were dissociated with a needle and syringe to expose both apical and basolateral surfaces to infection with MAdV-1 or MAdV-2. The mixture was then re-embedded in Matrigel extracellular matrix and cultured over 8 d. Virus released into the supernatant was titered on CMT-93 cells to quantify viral replication. Small intestinal enteroids supported the growth of MAdV-2, as evidenced by a >3-log increase in levels of progeny MAdV-2, which appeared to plateau around day 3 post-infection (Fig 1A). Although detectable levels of MAdV-1 were observed over 8 d in culture, no increase in infection was observed over this timeframe, leading to the conclusion that MAdV-1 was unable to replicate in small intestinal enteroids (Fig 1A). MAdV-1 was also unable to replicate in *Mmp7*^***-/-***^ enteroids. These results were somewhat surprising, since MAdV-1 reached higher titers than MAdV-2 in the mouse rectal cell line CMT-93 (Fig 1B). Due to the absence of MAdV-1 replication in enteroids, we used MAdV-2 for all subsequent experiments.

To visualize and quantify infection without immunostaining, we created a replication competent MAdV-2 expressing enhanced green fluorescent protein fused to the minor capsid protein IX (MAdV-2.IXeGFP). The design of this virus is similar to a previously described MAdV-1.IXeGFP [10]. MAdV-2 and MAdV-2.IXeGFP grow with similar kinetics in CMT-93 cells, although MAdV-2.IXeGFP does not replicate to the same levels as MAdV-2 (Fig 1B).

### Naturally secreted α-defensins enhance MAdV-2 infection of enteroids

**Fig 2:**
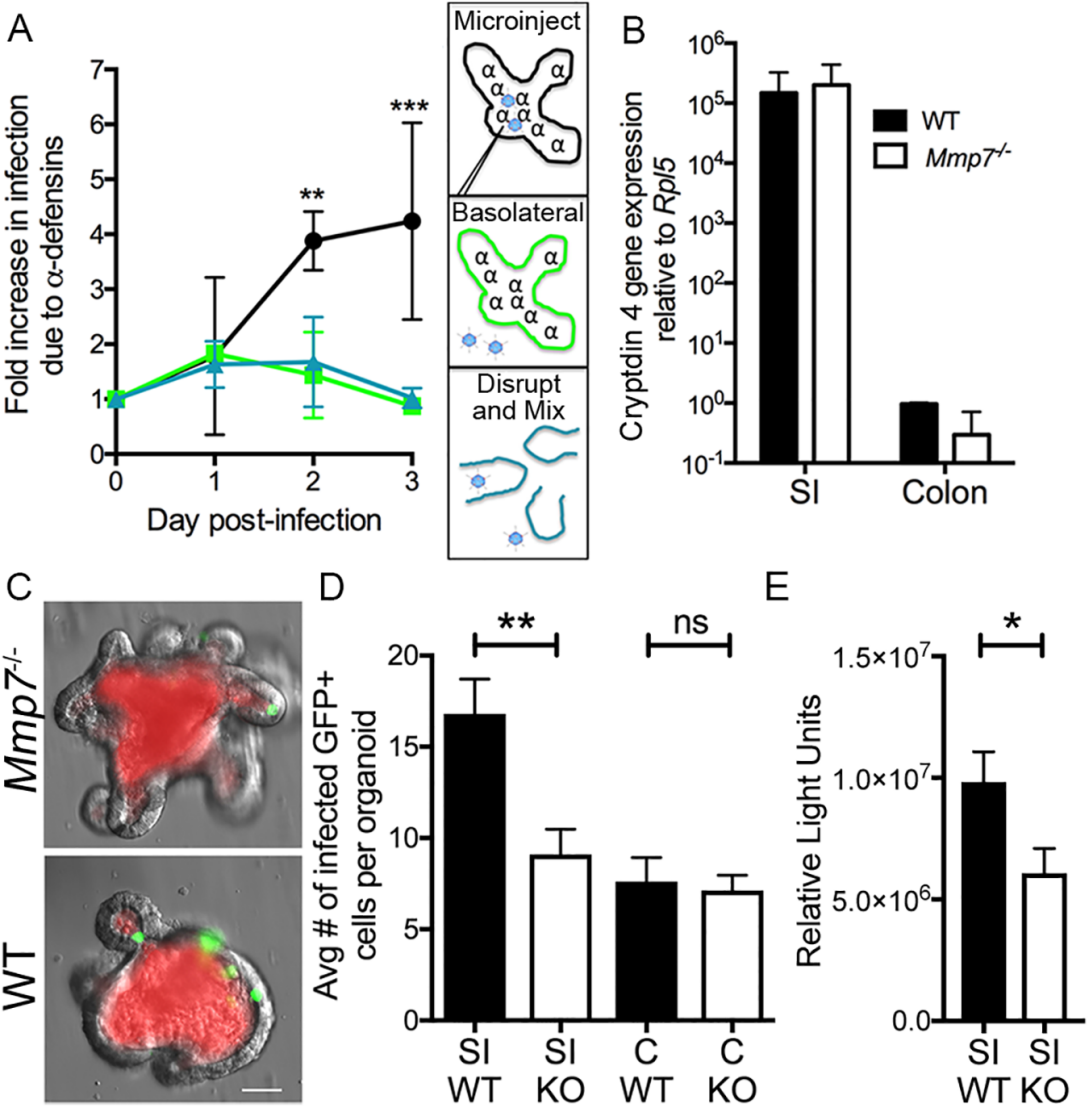
Naturally secreted α-defensins enhance MAdV-2 infection of small intestinal enteroids. (A) Wild type and *Mmp7*^***-/-***^ enteroids were infected with MAdV-2.IXeGFP via microinjection (circles, black line), basolateral infection (squares, green line), or disruption and mixing (triangles, blue line). The anticipated location of α-defensins (α) and virus (blue) relative to the enteroid cell layer (solid lines) are depicted for each route of infection in the schematics on the right. Viral titers from samples harvested on the indicated days were determined on CMT-93 cells. Data is the relative titer of progeny virus from wild type enteroids compared to *Mmp7*^***-/-***^ enteroids from 3 independent experiments ± SD. (B) Expression of cryptdin 4 (*Defcr4*) relative to expression of ribosomal protein L5 (*Rpl5*) in wild type and *Mmp7*^***-/-***^ enteroids from small intestine (SI) and colon was measured by qPCR four days after enteroid passage. Data are the average of two replicate experiments ± SD. (C) Representative images and (D) quantification of total GFP positive cells in wild type (WT, black columns) and *Mmp7*^***-/-***^ (KO, white columns) small intestinal (SI) and colonic (C) enteroids at 24 h post-infection with MAdV-2.IXeGFP by microinjection. Virus was mixed with Texas Red-conjugated dextran (red in C) to mark injected enteroids. Data is the average number of GFP positive cells from ten microinjected enteroids from at least 9 independent experiments ± SEM. (E) Relative light units of wild type and *Mmp7*^***-/-***^ small intestinal enteroids at 24 h post-infection with MAdV-2.IX2AFFluc by microinjection. Bars are colored as in (D). Data is the average of triplicate samples from 3 independent experiments ± SEM. **P<0.001; *P<0.05; ns, not significant.

To determine if naturally secreted α-defensins impact viral infection, we compared MAdV-2.IXeGFP infection of wild type enteroids, which secrete bactericidal concentrations of α-defensins, to that of *Mmp7*^***-/-***^ enteroids, which lack functional α-defensins [5]. We utilized microinjection to model natural apical infection via the enteroid lumen. In addition, enteroids were treated for 30 min prior to infection with the cholinergic agent carbamylcholine chloride (CCh), which induces Paneth cell degranulation [5], thereby maximizing α-defensin concentrations in the enteroid lumen. We infected parallel cultures, and a sample from each genotype was collected daily. Virus production was measured by titering the combined supernatant and lysate on CMT-93 cells. We observed equal progeny production on day 1; however, greater than 3-fold more virus was produced in wild type enteroids than in *Mmp7*^***-/-***^ enteroids on day 2 (Fig 2A). This increased to >4-fold more virus on day 3. Thus, rather than blocking infection, the presence of α-defensins enhanced the production of MAdV-2 in wild type enteroids.

Greater virus production in wild type enteroids could be explained by a substantial increase in initial infection or by a small cumulative effect of α-defensins on each round of replication and infection of new cells. To measure the number of initially infected cells for each genotype, we counted GFP positive cells in individual enteroids 24 h after microinjection. Given the ~20 h life cycle of adenovirus, GFP positive cells at this time point are exclusively from the initial infection. For this experiment, MAdV-2.IXeGFP was mixed with Texas Red-conjugated dextran in order to mark microinjected enteroids, and our analysis was restricted to enteroids that were still stained with Texas Red after 24 h (Fig 2C). Overall, approximately1.6-fold as many infected cells were observed in wild type enteroids compared to *Mmp7*^***-/-***^ enteroids (Fig 2C and 2D). To confirm that this effect was not due to the presence of the protein IX-GFP fusion protein, this experiment was repeated using a replicating MAdV-2 expressing firefly luciferase (MAdV-2.IX2AFFluc). The presence of a 2A sequence from porcine teschovirus-1 between the protein IX and firefly luciferase coding regions prevents the formation of a fusion protein (S1 Fig) [11]. Thus, only wild type protein IX is made in infected cells and incorporated into virus. We obtained a nearly identical 1.6-fold increase in infection of wild type compared to *Mmp7*^***-/-***^ enteroids (Fig 2E). Together, this data suggests that the major effect of enhancement by α-defensins in the wild type enteroids is due to an initial increase in infected cells.

In these cultures, deletion of MMP7 is not limited to Paneth cells. To rule out an effect of MMP7 other than α-defensin activation, we used two approaches. First, we compared MAdV-2.IXeGFP infection by two additional routes: basolateral, which bypasses the luminal α-defensins, and enteroid disruption and washing, which disperses the α-defensins. Under both of these conditions, we observed no difference in infection levels between genotypes (Fig 2A), confirming that small intestinal cells of both genotypes are equally able to support MAdV-2 infection and replication. Assuming that MAdV-2 progeny release from polarized cells is not directional (e.g., apical), these results also support our previous conclusion that α-defensin-dependent enhancement primarily acts on initial infection, since α-defensins in the lumen are only initially bypassed after basolateral infection or by mixing. As a second approach, we enumerated infected cells in colonic enteroids. Like the colon, colonic enteroids lack α-defensin expression (Fig 2B) due to the absence of Paneth cells. Unlike our results in small intestinal enteroids, no difference in the number of initially infected cells was seen between wild type and *Mmp7*^***-/-***^ colonic enteroids (Fig 2D). Moreover, the number of infected cells in colonic enteroids and *Mmp7*^***-/-***^ small intestinal enteroids was equivalent; indicating that the increase in infected cells observed in wild type small intestinal enteroids was due exclusively to α-defensins.

### Functional α-defensins increase MAdV-2 shedding *in vivo*

**Fig 3:**
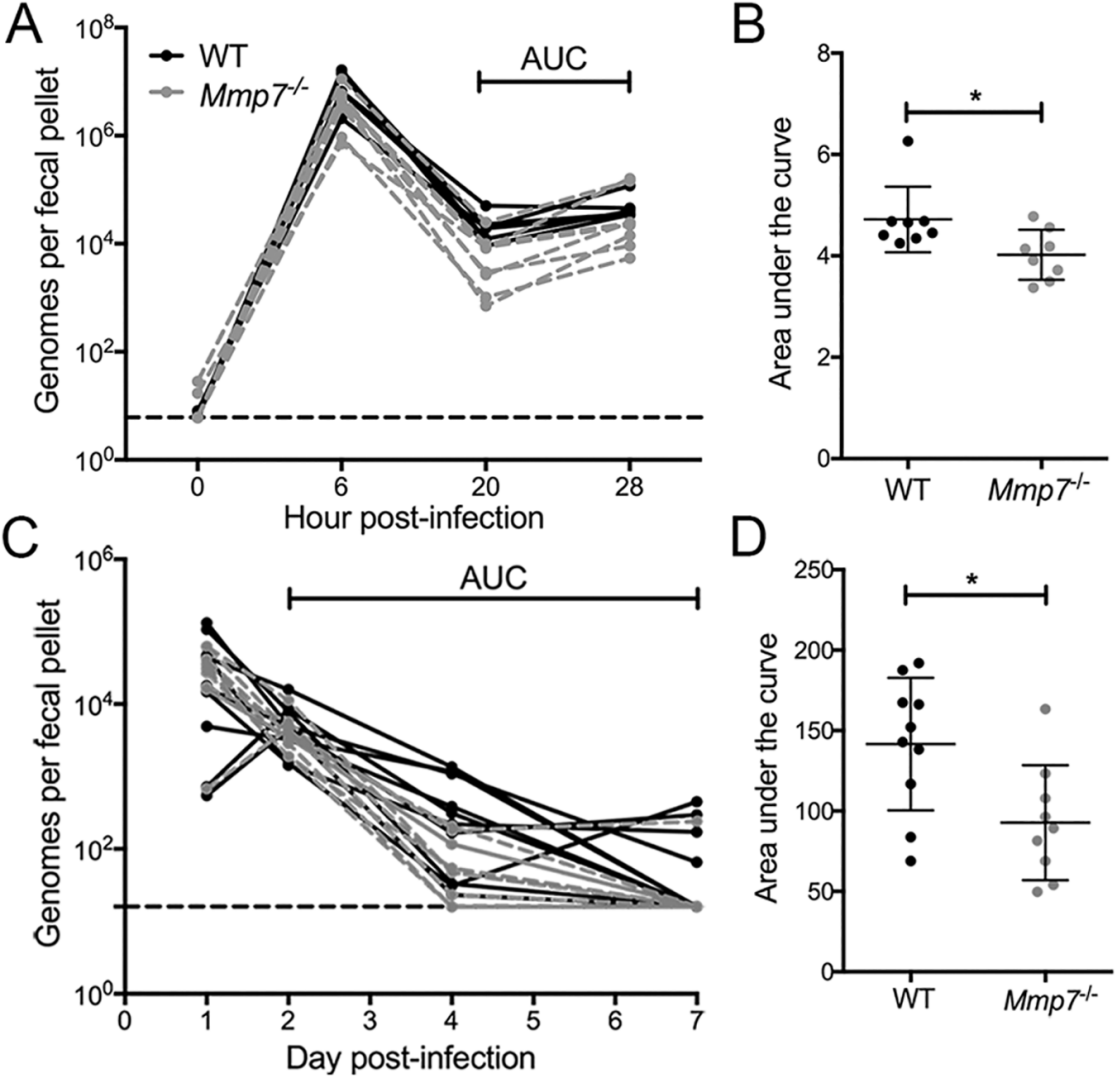
Fecal shedding of MAdV-2 is increased in mice expressing functional enteric α-defensins. Data are viral genomes per fecal pellet at the indicated times post infection for each wild type (solid black lines) or *Mmp7*^***-/-***^ mouse (grey dashed lines) after oral infection with (A and B) 1x10^7^ infectious units/mouse or (C and D) 1x10^6^ infectious units/mouse of wild type MAdV-2. Dashed black line in A and C indicates the limit of detection. (B and D) Total virus shed per mouse was calculated by log-transforming the data and determining the area under the curve (AUC) for the time range indicated in A and C for each mouse. N = 7–10 mice per group. In scatter plots, lines are mean ± SD. *P<0.05.

To determine if α-defensin-dependent viral enhancement also occurs *in vivo*, wild type and *Mmp7*^***-/-***^ mice were infected orally with 1x10^7^ iu/mouse of MAdV-2. Since MAdV-2 causes no overt pathology, infection and replication were monitored by quantifying fecal shedding at 6, 20, and 28 h post-infection. We observed a peak of shedding at 6 h, which likely represents passage of the inoculum. Between 20 and 28 h post-infection, wild type mice shed significantly more virus than *Mmp7*^***-/-***^ mice (Fig 3A and 3B). We then repeated this experiment over a longer time course. Wild type and *Mmp7*^***-/-***^ mice were infected orally with 1x10^6^ iu/mouse of MAdV-2, and fecal shedding was monitored at 2, 4, and 7 d post-infection. As in the previous experiment, wild type mice shed more virus than *Mmp7*^***-/-***^ mice (Fig 3C and 3D). Therefore, α-defensin-mediated enhancement of MAdV-2 infection results in increased viral shedding *in vivo* on a scale comparable to increased virus production observed in enteroids.

### Purified mouse enteric α-defensins bind to and enhance MAdV-2 infection in cell culture

**Fig 4:**
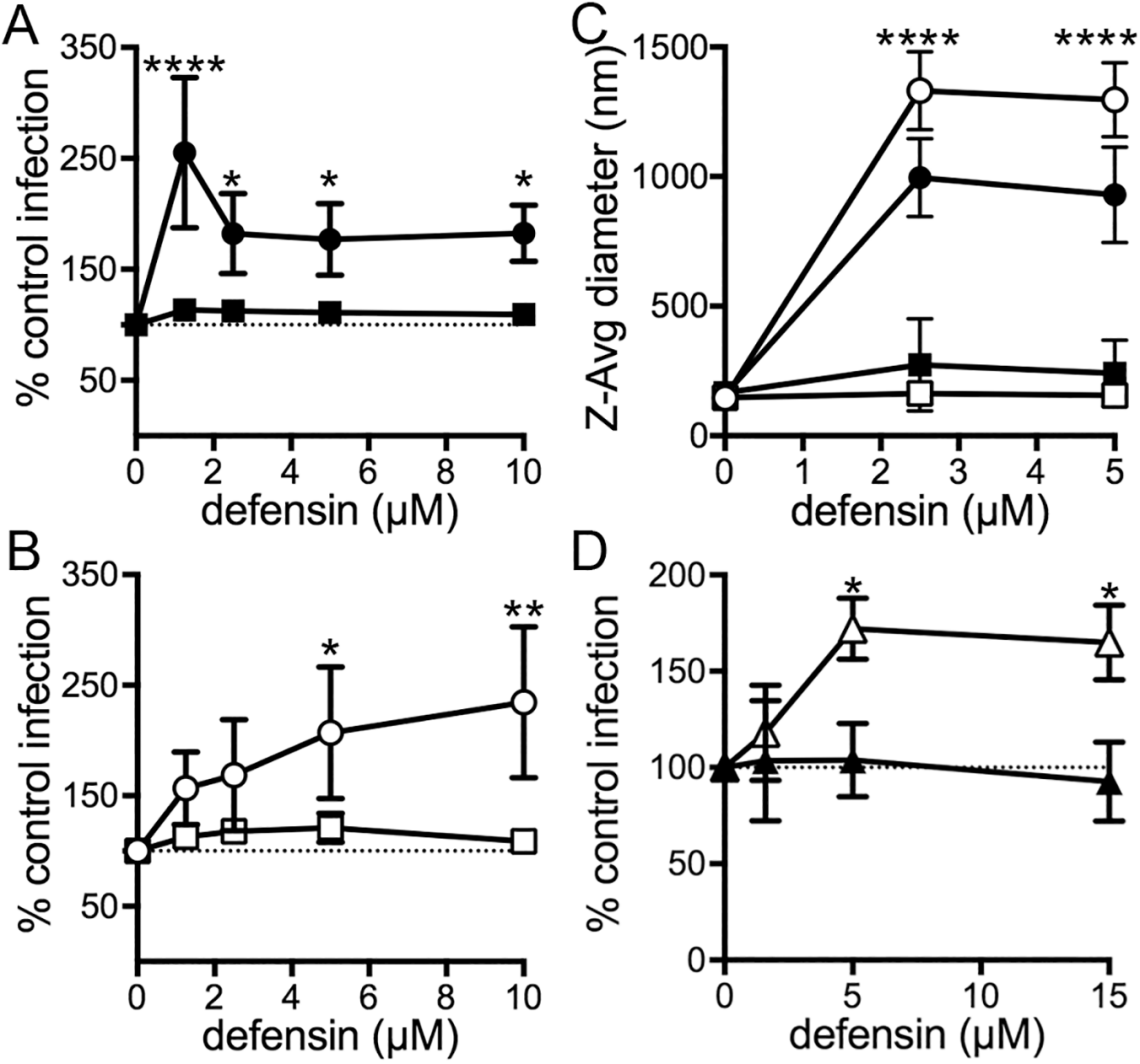
Purified enteric defensins but not pro-defensins bind to and enhance infection by MAdV-2 in cell culture. CMT-93 cells were infected with (A) MAdV-2 or (B) MAdV-2.IXeGFP that was pre-incubated with the indicated concentrations of the α-defensin cryptdin 2 (circles) or pro-cryptdin 2 (squares). Data is expressed relative to control cells infected in the absence of α-defensin (100%) and are the means of at least three independent experiments ± SD. *P<0.05, **P<0.01, ****P<0.0001 comparing cryptdin 2 to pro-cryptdin 2 at each concentration. (C) The z-average diameter of MAdV-2 (closed symbols) or MAdV-2.IXeGFP (open symbols) upon incubation with the indicated concentrations of cryptdin 2 (circles) or pro-cryptdin 2 (squares) was generated from cumulant analysis of dynamic light scattering. Results are the means of three independent experiments ± SD. ****P<0.0001 applies to both viruses when comparing cryptdin 2 to pro-cryptdin 2 at each concentration. (D) CMT-93 cells were infected with MAdV-2.IXeGFP that was pre-incubated with the indicated concentrations of the α-defensin cryptdin 3 (open triangles) or cryptdin 4 (closed triangles). Data is expressed relative to control cells infected in the absence of α-defensin (100%). Results are the means of at least three independent experiments ± SD. *P<0.05 relative to no defensin control.

To gain some insight into the mechanism of enhancement, we determined if purified α-defensins could enhance MAdV-2 infection in cell culture. We first compared MAdV-2 and MAdV-2.IXeGFP infection in the presence of cryptdin 2, one of the more abundant cryptdins *in vivo* [12]. Enhancement of infection for both viruses was dose-responsive (Fig 4A and 4B), although the effect of cryptdin 2 was greater for MAdV-2.IXeGFP (~2.4-fold) than wild type MAdV-2 (~1.8-fold). Importantly, the unprocessed pro-form of cryptdin 2 expressed in *Mmp7*^***-/-***^ mice had no effect on infection of either virus.

Enhanced infection suggested a direct interaction between the virus and α-defensins. To test this, we measured aggregation as a correlate of binding [13] and found that all cryptdin 2 concentrations tested led to an increase in the average z-diameter of both viruses, consistent with defensin binding to the viruses, while pro-cryptdin 2 did not bind to either virus (Fig 4C). Although the dose-responsiveness of binding to the two viruses was similar, larger aggregates of MAdV-2.IXeGFP than MAdV-2 formed in the presence of cryptdin 2. Therefore, defensin binds to the WT capsid and enhances infection; however, the fusion of GFP to protein IX may present additional surface area for defensin binding that increases both the degree of enhancement and the size of viral aggregates. Nonetheless, fusion of GFP to protein IX is not sufficient for enhanced infection, since MAdV-1.IXeGFP infection is neutralized by cryptdin 2 [14].

Because mice express multiple enteric α-defensin paralogs, we tested the activity of additional purified cryptdins on MAdV-2.IXeGFP infection. Cryptdin 3 increased infection ~1.7-fold, while incubation with cryptdin 4 had no effect on infection (Fig 4D). Thus, not all cryptdin paralogs are equally effective. Interestingly, we previously found that cryptdin 2 but not cryptdin 4 blocked infection by MAdV-1, and we attribute the inactivity of cryptdin 4 for both viruses to a three amino acid deletion that is not found in other cryptdins [14].

### Enteric α-defensins allow MAdV-2 to enter cells in a receptor-independent manner

**Fig 5:**
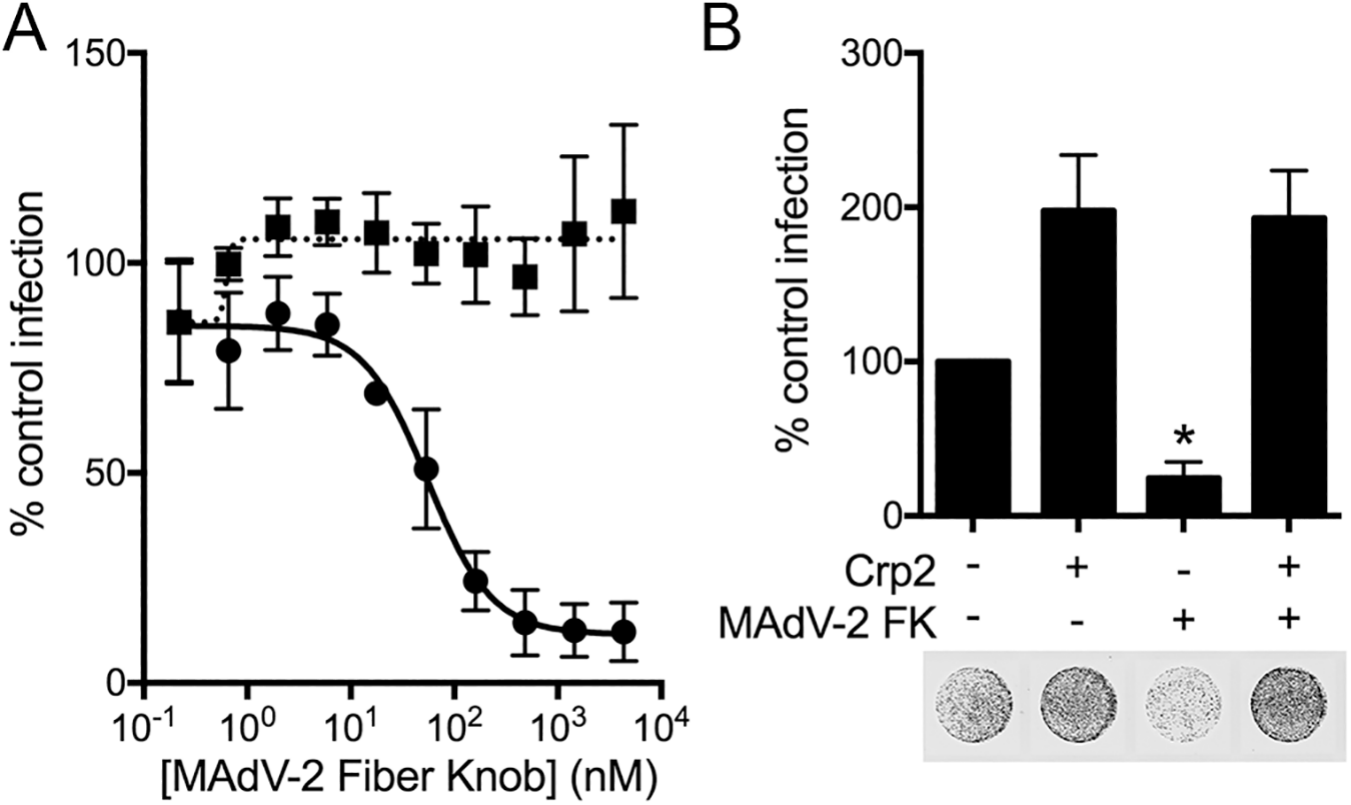
α-defensins allow MAdV-2 entry in a receptor-independent manner. (A) MAdV-2.IXeGFP (circles, solid line) or MAdV-1.IXeGFP (squares, dotted line) was added to CMT-93 cells pretreated with the indicated concentrations of MAdV-2 fiber knob, and infection was quantified 48 h post-infection. Results are the mean of two independent experiments ± SD. (B) MAdV-2.IXeGFP was pre-incubated with 5 μM cryptdin 2 (Crp2) or left untreated and then added to CMT-93 cells that had been pre-treated with 1.0 μM MAdV-2 fiber knob (FK) or left untreated. Infection was quantified 48 h post-infection. Data is expressed relative to control cells infected in the absence of α-defensin or FK (100%). Results are the mean of four independent experiments ± SD. Representative cell monolayers in 96 well plates were imaged at a resolution of 50 μm. Grayscale intensity correlates with eGFP expression. *P<0.05 relative to control.

We previously found that an interaction with the human enteric α-defensin HD5 increases the amount of HAdV bound to cells, despite the ability of HD5 to block endosome escape of internalized HAdV [4, 13]. Intriguingly, HD5-enhanced binding of HAdV to cells was independent of HAdV binding to its primary receptor, which could be blocked by outcompeting the virus with a molar excess of its receptor-binding domain, the fiber knob (FK) [4]. We speculated that cryptdins might enhance MAdV-2 infection by increasing virus binding to the cells but failing to block a downstream step in entry. To investigate this possibility, we first purified a recombinant construct consisting of the MAdV-2 FK and part of the distal shaft (residues 517–787) and verified its ability to specifically inhibit MAdV-2 but not MAdV-1 infection (Fig 5A). We then identified a minimal concentration of FK (1.0 μM) that could maximally (~84%) block MAdV-2.IXeGFP infection of CMT-93 cells in the absence of α-defensin. When MAdV-2.IXeGFP was pre-incubated with an enhancing α-defensin (cryptdin 2) before being added to cells pretreated with 1.0 μM MAdV-2 FK, we observed the same amount of enhancement as in the absence of MAdV-2 FK (Fig 5B). Taken together, these results indicate that the presence of α-defensins allows MAdV-2 to enter cells in a receptor-independent fashion. Moreover, since the degree of enhancement was equal in the presence and absence of FK, receptor-independent binding to cells is likely the major mechanism of enhanced infection.

## Discussion

We have uncovered a new means to promote enteric viral infection that is mediated by an effector of the host innate immune system, α-defensins, and is distinct from previously described mechanisms involving the intestinal microbiota or bile [15–19]. Since α-defensins are highly expressed at the site of natural MAdV-2 infection, our findings suggest that the virus has evolved to hijack these host-derived peptides to increase its own infection. For both mouse and human AdVs, serotypes with primarily gastrointestinal tropism (e.g., MAdV-2 and HAdV-F) are resistant to or enhanced by enteric α-defensins, while closely related, non-enteric serotypes (e.g., MAdV-1 and HAdV-C) are neutralized by α-defensins [4]. This trend is also consistent with some of the earliest observations regarding the antiviral activity of these proteins: echovirus and reovirus are enteric viruses that were found to be unaffected by α-defensins, establishing a dogma in the field regarding a general inability of α-defensins to neutralize non-enveloped viruses that was only recently dispelled [20, 21]. Thus, α-defensins are primarily antiviral, but we propose that certain non-enveloped viruses have evolved to become selectively resistant to and even enhanced by enteric α-defensins due to pressure imposed during fecal-oral transmission.

The closest equivalents to α-defensin enhancement of viral infection are antibody-dependent enhancement (ADE) of viruses (e.g., dengue) and increased uptake of viruses such as HIV-1 or West Nile Virus (WNV) that are opsonized with complement or lectins [22–25]. No other examples of enhancement of non-enveloped viral infection by α-defensins have been reported; however, HIV attachment and infection is increased by HD5 and HD6 [26, 27]. Although other innate immune effectors such as cytokines can increase viral infection, including adenovirus infection, their effects on infectivity have been indirect by modulating receptor distribution [28]. In contrast, ADE is mediated by an adaptive immune effector binding directly to the viral capsid, allowing viral entry through Fc or complement receptors [22]. It has been proposed that the divergence of dengue serotypes is driven by ADE [29, 30]. Similarly, the selective enhancement of enteric AdVs by enteric α-defensins suggests that this is an evolutionary adaptation of the virus to the intestinal milieu.

Unlike ADE, where the mechanism of enhancement is well understood, the precise mechanism of α-defensin enhancement remains to be determined. Since α-defensins are highly cationic, MAdV-2 enhancement may simply be due to charge neutralization allowing more virus to bind to the cell in the presence of α-defensins, similar to the effects of polybrene or other cationic compounds [2]. Indeed, we and others have documented increased cell binding due to α-defensins even for viruses that are subsequently neutralized [4, 13, 31]. Another possibility is that α-defensins bound to MAdV-2 facilitate an interaction with an alternate, defensin-specific cell surface receptor. α-defensins overcome the effects of a soluble competitor for primary receptor binding, suggesting receptor-independent entry of MAdV-2. This concept is difficult to test directly, as the cellular receptors for MAdV-2 and enteric α-defensins are unknown [32–35]. In ADE, interaction of the virus-antibody complex leads to Fcγ receptor- or complement-mediated entry into cells independent of dengue receptors, thereby expanding the range of cells susceptible to infection [22]. Similarly, a mosquito C-type lectin has been shown to enhance WNV infection of mosquitoes through interactions with a mosquito homolog of CD45 [25]. And, enhancement of HIV-1 infection by human α-defensins HD5 and HD6 is also independent of CD4 and co-receptors [26]. For HAdVs in particular, an analogous mechanism governs liver and spleen cell tropism via blood coagulation factors that bind to hexon and bridge interactions with heparin sulfate proteoglycans on the cell surface [36]. Alternatively, some mouse α-defensins can permeabilize eukaryotic membranes [37], perhaps leading to increased infection by forming pores at the cell surface or facilitating disruption of the endosomal membrane, resulting in more virus reaching the nucleus. Since α-defensins bind to both enhanced and neutralized HAdV and MAdV serotypes and therefore enter the endosomal pathway [4, 13, 14, 38, 39], they may remain bound to and prevent uncoating of neutralized AdVs but dissociate from enhanced AdVs to interact with the endosomal membrane. Although our data provide the strongest support for α-defensins impacting cell binding, enhanced infection could be explained by facilitating internalization. Complement opsonized HIV-1 uptake by dendritic cells is enhanced in this manner [24]. We found no alteration in internalization kinetics in our studies of neutralization of HAdVs by α-defensins [38]; however, this has not yet been investigated for enhanced infection. Defensins could also act post-entry by promoting uncoating of enhanced viruses, perhaps by destabilizing the internalized capsid in a manner analogous to their activity against bacterial toxins [40]. Irrespective of the specific mechanism, our results suggest that the distribution of α-defensins in the gastrointestinal tract may dictate the location of enteric viral infections.

MAdV-2 infection is enhanced by α-defensins in three different systems: upon addition of purified α-defensins to two-dimensional cell culture *in vitro*, due to naturally secreted α-defensins in small intestinal enteroids, and *in vivo*. While there has been much interest in intestinal enteroids as a novel model for the human and mouse intestinal tract, there is a need to determine whether enteroids are truly predictive of *in vivo* effects in a manner distinct from traditional cell culture systems. We demonstrate increased viral replication specific to wild type small intestinal enteroids, which is not observed in colonic enteroids or *Mmp7*^***-/-***^ enteroids. These results were recapitulated *in vivo*, since orally infected wild type mice shed more virus than *Mmp7*^***-/-***^ mice in multiple experiments. Therefore, our data as well as other recently published papers support the idea that mouse enteroids are highly predictive of *in vivo* effects [41–43]. These studies also imply that results from human intestinal enteroids would be directly applicable to the human intestine. As human enteroids are a renewable source of primary cells, they represent an unprecedented opportunity for experimentation in a system that more accurately reflects human biology than cell lines or animal testing.

## Materials and methods

### Ethics statement

All mouse experiments were performed in strict accordance with the Guide for the Care and Use of Laboratory Animals of the National Institutes of Health and following the International Guiding Principles for Biomedical Research Involving Animals. Animals were humanely euthanized by CO_2_ inhalation for infection studies or by bilateral thoracotomy under anesthesia to harvest tissue for enteroid culture. Protocols were approved by the Institutional Animal Care and Use Committee of the University of Washington under Protocol Number 4245–01.

### Enteroid culture

Small intestinal enteroids were derived from crypt enriched ileal fractions from 6–10 week old wild type and *Mmp7*^***-/-***^ mice on a C57BL/6NHsd background as previously described [5] and maintained in Complete Crypt Culture Medium (CCCM) [44]. Colonic enteroids were similarly derived and cultured from crypt enriched colonic fractions with the following modifications: 1) To avoid adherence of colonic enteroids during derivation, plastic ware was pretreated with 10% FBS. 2) Pelleted crypts were treated with TrypLE (Thermo Fisher Scientific) for 5 min at 37°C prior to washing and embedding in Matrigel. 3) CCCM was supplemented with murine Wnt-3A (0.1 μg/mL, Sigma-Aldrich), CHIR99021 (2.5 μM final, Stemcell Technologies), and valproic acid (1 mM, Sigma-Aldrich). Once enteroids were established, culture media was supplemented with 200 μl CCCM every 2–3 days. Enteroids were subcultured every 6–7 days [44].

### Viruses

Wild type MAdV-2 was a gift from Susan Compton (Yale University School of Medicine). MAdV-1 and MAdV-1.IXeGFP were gifts from Katherine Spindler (University of Michigan) [10]. Replication-competent MAdV-2.IXeGFP was patterned on MAdV-1.IXeGFP and produced using recombineering with a bacterial artificial chromosome containing the genome of MAdV-2 (pKSB2 MAdV-2, a kind gift from Silvio Hemmi, University of Zurich) [45]. MAdV-2.IX2AFFluc was similarly constructed by inserting a sequence encoding the 2A peptide from porcine teschovirus-1 (AGATNFSLLKQAGDVEENPGPAAA) [11] followed by a sequence encoding firefly luciferase immediately before the stop codon of protein IX by recombineering. All MAdVs were propagated in CMT-93 mouse rectal carcinoma cells (ATCC CCL-223) and purified as described [14]. To quantify virus production in Fig 1B, multiple wells of CMT-93 cells were infected in parallel with MAdV-2, MAdV-2.IXeGFP, or MAdV-1 at an MOI of 3. Beginning on the day of infection, cells and supernatant were collected from one well daily for 5 days. After freeze/thaw and centrifugation, lysates were serially diluted on a fresh monolayer of CMT-93 cells. Infection levels were quantified 48 h post-infection by staining fixed cells with anti-hexon antibody 8C4 and an Alexa Fluor 488-conjugated secondary antibody. Total well fluorescence was quantified with a Typhoon 9400 scanner. For each virus at each time point, the TCID_50_ was determined and used to calculate FFU/mL based on the Poisson distribution.

### Enteroid viral infection assays

For infection by mixing in Figs 1A and 2A, enteroids were subcultured prior to infection and overlaid with 500 μL CCCM in a 24-well tissue culture dish. After 3–4 days, enteroids in Matrigel plugs from 6 wells were pooled and disrupted with a pipette tip, and the suspension was dissociated with a 25-gauge needle. Cells were then pelleted by pulse centrifugation in a mini-centrifuge and resuspended in 250 μl CCCM. MAdV-2.IXeGFP, MAdV-1, or MAdV-2 (1-2x10^5^ infectious units) in 250 μl of CCCM was added to the cells and incubated at 4°C for 30 min to allow for virus binding. Cells were then washed twice in CCCM to remove unbound virus and resuspended in 30 μl CCCM and 120 μl Matrigel. 30 μl was plated into each of 5 wells of a 24 well plate, allowed to polymerize, and then overlaid with 500 μl CCCM.

For basolateral infections, media was removed from 2–3 day old enteroids seeded in 24-well plates and replaced with 500 μl of fresh CCCM containing MAdV-2.IXeGFP (1-2x10^5^ infectious units).

For infection by microinjection, enteroids were cultured in Matrigel on glass coverslips. Prior to injection, cells were treated with 10 μM CCh (Sigma-Aldrich) for 30 min. Ten enteroids per coverslip were injected with 200–250 pL per enteroid of MAdV-2.IXeGFP (4x10^8^ infectious units/mL) alone or mixed 1:1 with 80,000 MW Texas Red-conjugated dextran using the same needle for all wild type and *Mmp7*^***-/-***^ enteroids from a single experiment. After injection, wells were washed twice and overlaid with 1 mL CCCM. For Fig 2C and 2D infected (GFP positive) cells per injected enteroid (Texas Red-conjugated dextran positive) were manually counted using an epifluorescence microscope. For Fig 2E, fifteen enteroids of each genotype were injected with 200–250 pL per enteroid of MAdV-2.IX2AFFluc from a virus stock with a concentration of 3.6x10^11^ genomes/mL. Enteroids were lysed 24 h post-infection, and luciferase activity was measured using the Promega Dual Luciferase Reporter assay.

For Figs 1A and 2A, samples of combined cells and supernatant were collected immediately after washing (day 0) or daily for 8 or 3 days respectively. The combined cells and supernatant underwent three rounds of freeze/thaw to release intracellular virus. After centrifugation, 50 μl of the virus-containing supernatant was applied to a fresh monolayer of CMT-93 cells. Infection levels were quantified by staining for hexon as described above. Data are the integrated density of total well fluorescence (Fig 1A) or the ratio of total well fluorescence of monolayers infected with progeny from wild type compared to *Mmp7*^***-/-***^ enteroids (Fig 2A).

### Gene expression analysis

Total RNA was extracted from enteroids after 4 days in culture using RNAbee (Tel-Test, Inc.). RNA was primed with Oligo DT and reverse transcribed. qPCR was performed using the primers listed in Table 1. *Defcr4* expression was calculated relative to the housekeeping gene *Rpl5*.

**Table 1:** Primers used.

Name	Sequence
RPL5 F	5’- GGAAGCACATCATGGGTCAGA -3’
RPL5 R	5’- TACGCATCTTCATCTTCCTCCATT -3’
Defcr4 F	5’- CCAGGGGAAGATGACCAGGCTG -3’
Defcr4 R	5’- TGCAGCGACGATTTCTACAAAGGC -3’
M2FF2	5’- GTCCGATTCGGTACTACGGT -3’
M2FR2	5’- GTCAGACAACTTCCCAGGGT -3’

### Mouse infections and fecal shedding

Mice were housed under specific pathogen-free conditions and ABSL-2 containment and were infected between 5 and 7 weeks of age via oral gavage with purified wild type MAdV-2 diluted in sterile PBS. For experiments in Fig 3A and 3B, mice were housed 5 per cage and segregated by genotype. Fresh fecal pellets were collected beginning immediately before infection and at 6, 20, and 28 h post-infection. For the experiments in Fig 3C and 3D, mice were individually housed beginning 3 or 4 d prior to infection and for the duration of the experiment. Fecal samples consisted of ten fecal pellets that accumulated in the cages since the previous collection. Accordingly, on the day of infection and after every fecal collection, mice were transferred to new cages with clean bedding.

Viral DNA was extracted from fecal pellets using the QIAamp DNA Stool Mini Kit (Qiagen) into a total volume of 200 μl. MAdV-2 genomes were quantified by qPCR against a standard curve of pKSB2-MAdV2 using primers M2FF2 and M2FR2 (Table 1). Reaction conditions consisted of 40 cycles of PCR with 55°C annealing temperatures. The limit of detection was defined by the number of viral copies detected in feces from uninfected mice.

### Purified defensin and fiber knob production

Cryptdin 2 (UniProtKB: P28309, LRDLVCYCRTRGCKRRERMNGTCRKGHLMYTLCCR) and pro-cryptdin 2 (DPIQNTDEETKTEEQSGEEDQAVSVSFGDREGASLQEESLRDLVCYCRTRGCKRRERMNGTCRKGHLMYTLCCR) were obtained by oxidative refolding of partially purified linear peptides (synthesized by CPC Scientific and LifeTein, respectively) and purifying the correctly folded species by reverse-phase high-pressure liquid chromatography (RP-HPLC). Purity was determined by analytical RP-HPLC, and the mass of the disulfide-bonded peptides was verified by high mass accuracy liquid chromatography-mass spectrometry. Additional cryptdin 2 was a gift from Wuyuan Lu (University of Maryland). Cryptdin 3 (UniprotKB: P28310, LRDLVCYCRKRGCKRRERMNGTCRKGHLMYTLCCR) and Cryptdin 4 (UniprotKB: P28311, GLLCYCRKGHCKRGERVRGTCGIRFLYCCPRR) were a gift from André Ouellette (University of Southern California). All α-defensin concentrations were quantified by UV absorbance at 280 nm using calculated molar extinction coefficients.

A recombinant protein containing the MAdV-2 fiber knob and ~6.5 distal shaft repeats was generated from a bacterial expression plasmid (pET28(+)) encoding MAdV-2 residues 517–787 with an N-terminal 6× His tag (a gift from Mark J van Raaij, Spanish National Center for Biotechnology). Protein was expressed in BL21-CodonPlus (DE3)-RIPL cells (Agilent Technologies) upon induction with 0.4 mM IPTG for 4 hr at 37°C; purified using a TALON column (Clontech), as previously described [46]; and stored in 50 mM Na_2_HPO_4_/NaH_2_PO_4_, 130 mM NaCl, 10% glycerol, pH 8.0.

### Inhibition assays

Dilutions of MAdV-2 or MADV-2.IXeGFP producing ~50% maximal signal 48 h post-infection of CMT-93 cells were chosen for inhibition studies. Defensin studies were performed as described with purified virus in serum-free DMEM (SFM) [14]. For Fig 5A, increasing concentrations of MAdV-2 fiber knob were mixed with virus in SFM in a total volume of 50 μl, added to cells, and incubated at 37°C for 2 h. Cells were then washed and cultured with DMEM containing 10% FBS for 48 h prior to quantification. For experiments in Fig 5B, virus was pre-incubated with a final concentration of 5 μM cryptdin 2 in a total volume of 37.5 μl on ice. In parallel, a confluent monolayer of CMT-93 cells was pre-treated at 37°C with SFM containing 1 μM MAdV-2 fiber knob or an equal concentration of BSA in a final volume of 50 μl SFM. After 45 min, 12.5 μl of SFM containing 4 μM MAdV-2 fiber knob or BSA was added to the virus and defensin mixture. The pre-treated CMT-93 monolayer was washed twice with SFM, and 50 μl of the virus, defensin, and fiber knob or BSA mixture was added to cells and incubated for 2 h at 37°C. Cells were then washed and cultured with DMEM containing 10% FBS for 48 h prior to quantification.

### Dynamic light scattering

α-defensins were serially diluted in 10 mM Tris, 150 mM NaCl, pH 7.5 and mixed with 6.5x10^8^ particles of wild type MAdV-2 or MAdV-2.IXeGFP in a total volume of 50 μl. Control samples of virus or α-defensin only were diluted in the same buffer. Samples were incubated for 45 min on ice and then equilibrated for 3 min at 37°C prior to analysis. The z-average particle size was obtained by cumulant analysis with a Malvern Zetasizer Nano ZS and manufacturer’s software (Malvern Instruments).

### Statistics

Experiments were analyzed using Prism (v. 7.0b, GraphPad). For Fig 2A, 2-way ANOVA with Tukey post-test was used to compare the mean of each infection route with the mean of every other infection route for each time point. In Fig 2D, data was analyzed using one-way ANOVA comparing the means of wild type and *Mmp7*^***-/-***^ samples. In Fig 2E, relative light units of triplicate samples were averaged. The log-transformed averages from three independent experiments were then compared by paired, two-tailed t test. In Fig 3B and 3D, genome copy numbers were log transformed prior to determining total viral shedding by calculating the area under the curve (AUC) over the indicated time period. AUCs were compared by unpaired t test. In Fig 4A, 4B or 4C data for each virus in the presence of cryptdin 2 was compared to data in the presence of pro-cryptdin 2 at each concentration by two-way ANOVA with Sidak’s multiple comparison tests. In Figs 4D and 5B, infection for each defensin concentration or condition was compared to infection in the absence of defensin by one-way ANOVA with Dunnett’s multiple comparison test. *P<0.05, **P<0.001, ***P<0.0001

## Supporting information

S1 Fig Insertion of the porcine teschovirus-1 2A sequence between protein IX and luciferase precludes formation of a fusion protein. Lysates of cells infected with MAdV-2.IX2AFFluc (lanes 1 and 6, M2 2Aluc), MAdV-2.IXeGFP (lanes 2 and 7, M2 GFP), and HAdV-5.eGFP (lanes 3 and 8, H5 eGFP) and recombinant firefly luciferase (lanes 4 and 9, rLuc, Promega E1701) were probed by immunoblot for GFP and luciferase. The IX-GFP fusion (41 kDa) is clearly distinguished from GFP (30 kDa). In contrast, luciferase from the MAdV-2.IX2AFFluc construct has the same mobility as rLuc (62 kDa). There is no detectable band consistent with the predicted mobility of a IX-luciferase fusion protein (73 kDa). Thus, we conclude that there is no IX-luciferase fusion protein produced in infected cells that could then be incorporated into virus.
(PDF)
