# Supplementary material for: Alpha-defensin-dependent enhancement of enteric viral infection
Source: PLoS Pathog. 2017 Jun 16;13(6):e1006446. doi: 10.1371/journal.ppat.1006446 (PMC5489213; doi:10.1371/journal.ppat.1006446)
Supplement: S1 Fig — Lysates of cells infected with MAdV-2.IX2AFFluc (lanes 1 and 6, M2 2Aluc), MAdV-2.IXeGFP (lanes 2 and 7, M2 GFP), and HAdV-5.eGFP (lanes 3 and 8, H5 eGFP) and recombinant firefly luciferase (lanes 4 and 9, rLuc, Promega E1701) were probed by immunoblot for GFP and luciferase. The IX-GFP fusion (41 kDa) is clearly distinguished from GFP (30 kDa). In contrast, luciferase from the MAdV-2.IX2AFFluc construct has the same mobility as rLuc (62 kDa). There is no detectable band consistent with the predicted mobility of a IX-luciferase fusion protein (73 kDa). Thus, we conclude that there is no IX-luciferase fusion protein produced in infected cells that could then be incorporated into virus. (PDF) [file ppat.1006446.s001.pdf]

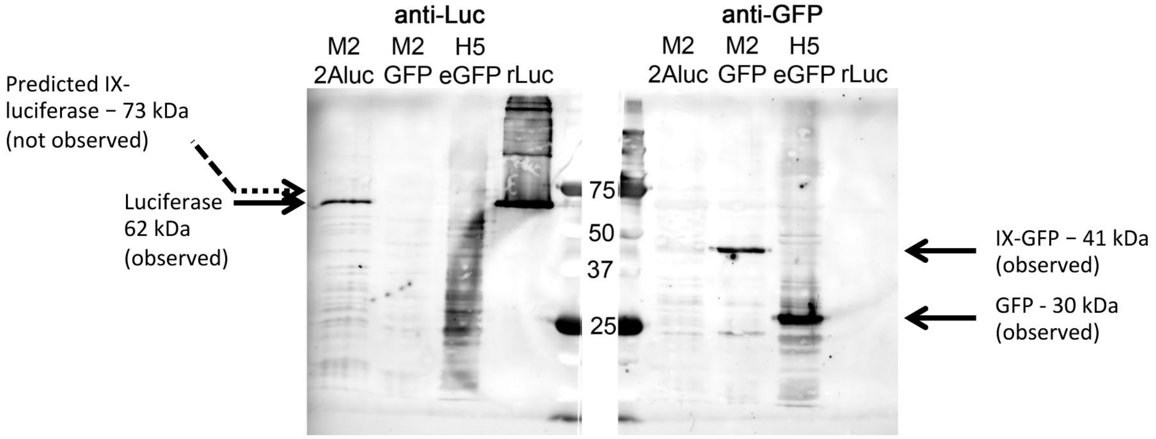


**S1 Fig. Insertion of the porcine teschovirus-1 2A sequence between protein IX and luciferase precludes formation of a fusion protein.** Lysates of cells infected with MAdV-2.IX2AFFluc (lanes 1 and 6, M2 2Aluc), MAdV-2.IXeGFP (lanes 2 and 7, M2 GFP), and HAdV-5.eGFP (lanes 3 and 8, H5 eGFP) and recombinant firefly luciferase (lanes 4 and 9, rLuc, Promega E1701) were probed by immunoblot for GFP and luciferase. The IX-GFP fusion (41 kDa) is clearly distinguished from GFP (30 kDa). In contrast, luciferase from the MAdV-2.IX2AFFluc construct has the same mobility as rLuc (62 kDa). There is no detectable band consistent with the predicted mobility of a IX-luciferase fusion protein (73 kDa). Thus, we conclude that there is no IX-luciferase fusion protein produced in infected cells that could then be incorporated into virus.
